# MED9/368: Health education over Internet: Is there a special way for smaller language groups?

**DOI:** 10.2196/jmir.1.suppl1.e52

**Published:** 1999-09-19

**Authors:** J Aru

**Affiliations:** ^1^Doctus, TartuEstonia

**Keywords:** Health Education, Health Promotion

## Abstract

Health education over resources, that use English, German and French language, is a rapidly growing area of medicine within the World Wide Web. Multiple gateways and portal servers have been set up to guide people within the diversity of materials available. For smaller language groups the situation is quite different: there is a lack of information in even the basic medical education topics. The question is: how to fill the missing gaps. Principally two different ways are possible:

1. Translating materials from English, German, French and other "big" language groups.
2. Generating original Internet-based texts for "smaller" language group

When using the first, possibility of problems related to copyright and proper translating occur. When using the second approach, the quality of materials could suffer. As an example, the homepage for health education www.doctus.ee is discussed in detail. This homepage includes materials about the health care system, basic health topics, main diseases and also a questionnaire for consulting one's health problems with a doctor. A year's experience running this health education Web site in Estonian has brought to following conclusions:

1. For translations a link for originals should be included.
2. With original Estonian texts copyright questions are frequent.
3. Guidance is frequently needed for translated texts.
4. Health consultations over Internet should be legally regulated

